# Work and Dialysis: Navigating Employment Challenges Among Young Adults on Maintenance Hemodialysis in Tanzania

**DOI:** 10.1155/ijne/9980961

**Published:** 2025-11-03

**Authors:** Daniel Msilanga, Upendo Nkwera, Priyank Punatar, Jonathan Mngumi, Elizabeth Msangi, Gudila Valentine, Happiness Mushi, Jacqueline Shoo

**Affiliations:** ^1^Department of Internal Medicine, Muhimbili University of Health and Allied Sciences, Dar es Salaam, Tanzania; ^2^Department of Internal Medicine, Muhimbili National Hospital, Dar es Salaam, Tanzania

## Abstract

**Introduction:**

Chronic kidney disease (CKD) increasingly affects young adults in low-and middle-income countries, with significant social and economic consequences. In Tanzania, many patients begin dialysis during their most productive years, often disrupting employment and financial stability. This study aimed to examine employment patterns and dialysis-related work challenges among young adults on maintenance hemodialysis at Muhimbili National Hospital.

**Methods:**

We conducted a hospital-based cross-sectional study at Muhimbili National Hospital from October to December 2024, involving adults aged 18–49 years on maintenance hemodialysis for at least three months. Data were collected using a structured questionnaire and supplemented with clinical records to assess sociodemographic, dialysis-related, employment, and transplant-related factors. Descriptive statistics were used to summarize the findings.

**Results:**

We included 134 patients aged between 18 and 49 years, with a mean age of 35.8 years (SD: 8.9). A total of 58.3% were either employed or self-employed, with 44.9% reporting that they did not work on dialysis days. Among those working, 65.4% experienced changes in their work patterns due to treatment demands, and 42.4% worked fewer than 4 days per week. Among the unemployed participants (24.6%), more than half (57.6%) reported resigning from work due to dialysis-related challenges. Out-of-pocket payment for dialysis was common, reported by 52.3% of participants. Although awareness of kidney transplantation was high (92.5%), only 27.6% had previously pursued or were actively pursuing it, primarily hindered by the absence of a donor (48.3%), high cost (23.0%), or both (28.7%).

**Conclusion:**

Young adults on maintenance hemodialysis in Tanzania face significant employment disruptions and limited access to kidney transplantation, highlighting the need for integrated interventions to support their social and economic well-being.

## 1. Introduction

Chronic kidney disease (CKD) is a rising global health burden, with a disproportionately high impact on low- and middle-income countries (LMICs) [1]. Unlike in high-income settings where CKD often affects older adults, in Sub-Saharan Africa and other similar regions, it tends to present at a much younger age [2]. In Tanzania, as in many LMICs, a growing number of young adults are developing CKD during their most economically productive years [3–5]. This early onset has serious social and economic consequences affecting not only individual patients but also their families, communities, and national economies due to the loss of manpower, productivity, and years of potential life [4].

The economic impact of CKD on working-age adults is far-reaching [4, 6]. Many patients are primary breadwinners, and their diagnosis often leads to job loss, reduced income, and rising out-of-pocket medical expenses [7]. This loss of financial independence increases dependence on family members and caregivers [8]. In addition, governments also bear indirect costs through reduced workforce participation and rising healthcare expenditures [6, 9, 10]. The burden is especially pronounced and long-lasting in settings where kidney transplantation remains largely inaccessible, as is the case in many African countries, where maintenance hemodialysis has become the primary form of renal replacement therapy [11, 12]. However, while lifesaving, this treatment is time-consuming, often incompatible with formal employment, and frequently associated with fatigue [13, 14]. Regular dialysis sessions, typically two to three times per week, not only interfere with standard working hours but also impose significant logistical and physical burdens on patients [7].

Tanzania has a population of approximately 60 million people, with individuals aged 18–49 years comprising nearly half of the population and representing the country's most productive workforce [15]. Despite this demographic advantage, employment challenges remain significant [16]. Nearly half of the working-age population is engaged in informal or self-employment, often without job security or social protection [15, 16]. Although national data on employment status among dialysis patients are limited, findings from several studies in Tanzania suggest that a significant proportion of patients despite being young, are unemployed during the course of their dialysis treatment [17, 18].

Despite the growing population of young adults on dialysis, little is known about how this therapy affects their daily lives, particularly in terms of employment among those without access to kidney transplantation. Existing literature from LMICs often emphasizes clinical outcomes, with limited data on socioeconomic impacts. This study seeks to address this gap by examining employment patterns and job-related challenges among young adults on maintenance hemodialysis at Muhimbili National Hospital (MNH) in Tanzania. By generating evidence on how CKD and dialysis affect working-age adults, this study will highlight the need for integrated support systems to assist this vulnerable population.

## 2. Methods

### 2.1. Study Settings and Recruitment Process

A hospital-based cross-sectional study was conducted at the MNH hemodialysis units from October to December 2024. MNH is the largest tertiary-level public health facility in Tanzania, with a capacity of 1500 beds and a dialysis unit equipped with 50 hemodialysis machines. The unit serves between 140 and 180 patients with CKD daily.

We included patients aged 18–49 years who had been on maintenance hemodialysis therapy for at least three months. Eligible participants were identified from the hospital's dialysis registry. The study objectives, procedures, risks, and benefits were explained to each patient, and those who provided written informed consent were included. This recruitment process continued until all eligible patients available during the study period were approached.

The sample size was calculated using a single population proportion formula with an assumed employment disruption prevalence of 50% among dialysis patients and a margin of error of 5%. The minimum sample size required was 125 participants [17, 19].

Data were collected using a structured, interviewer-administered questionnaire to capture sociodemographic information (age, sex, marital status, education level, smoking, and alcohol use), transportation methods, distance traveled to dialysis unit (estimated using Google Maps to calculate the road distance between the patient's residence and the hospital), and dialysis-related factors including escort to dialysis, frequency of sessions, duration on dialysis, dialysis payment modality (insurance or out-of-pocket), type of vascular access (arteriovenous fistula[AVF], tunneled, or nontunneled catheter), and distance from the dialysis center.

Clinical data such as the underlying cause of CKD were extracted from electronic medical records. Employment-related variables, current occupational status, ability to work on dialysis days, changes in work patterns, and dialysis-related job resignation, were obtained through self-report. In addition, information on kidney transplant awareness, current transplant work-up status, and barriers to transplantation (absence of a donor, high cost, or both) was documented.

### 2.2. Data Analysis

Descriptive statistics were used to summarize baseline demographic, clinical, and employment-related characteristics. Continuous variables were reported as means with standard deviations (SDs) or medians with interquartile ranges (IQRs), depending on data distribution. Categorical variables were presented as frequencies and percentages. Data analysis was performed using standard statistical software.

## 3. Results

A total of 134 patients were included in this study, with a mean age of 35.8 years (SD: 8.9). The majority were male (55.9%), single (53.7%), and had attained tertiary education (44.8%). Most participants reported no history of alcohol use (82.8%) or cigarette smoking (90.3%). The median distance to the dialysis facility was 12.5 km (IQR: 8.8–15.0), and public transportation was the most commonly used mode of travel (75.4%).

More than half (61.2%) attended dialysis sessions with an escort from home. In addition, 51.5% received hemodialysis twice weekly, and 56.0% had been on dialysis for less than 24 months, with a median of 19 (9.3–42). Hypertension was the leading cause of CKD (59.1%), followed by chronic glomerular diseases (18.2%). AVF was the most frequently used vascular access (38.1%), followed by tunneled catheters (35.1%) and nontunneled catheters (26.9%) (Table 1).

Among the study participants, 58.3% were either employed or self-employed, and 44.9% of them reported not working on the days they attended dialysis. Among those who were working, 65.4% experienced changes in their work patterns due to dialysis treatment with only about half reporting to work for more than 4 days a week (57.6). Of those who were unemployed (24.6%), more than half (57.6%) reported having resigned from work due to dialysis-related challenges. Nearly half of the patients (52.3%) paid out of pocket for dialysis. Awareness of kidney transplantation was high, with 92.5% of participants reporting knowledge of the procedure; however, only 27.6% reported a previous or active pursuit of kidney transplantation. Among the 87 participants who reported barriers to transplantation, 48.3% cited the absence of a donor, 23.0% identified high cost, and 28.7% reported both high cost and lack of a donor as obstacles (Table 2).

## 4. Discussion

Our study provides valuable insight into the impact of dialysis on the working lives of young adults on maintenance hemodialysis. We included 134 patients with a mean age of 35.8 years (SD: 8.9). More than half were employed or self-employed, yet nearly half did not work on dialysis days, and reported changes in their work routines due to treatment demands, leading to working less than 4 days per week. Among the unemployed, more than half had resigned because of dialysis-related challenges. Out-of-pocket dialysis payment was common, and while awareness of kidney transplantation was high, only a quarter had previously or actively pursued it, mainly hindered by the lack of a donor or the high cost.

A notable proportion of young and middle-aged patients on dialysis were able to sustain employment, either through formal jobs or self-employment. However, nearly half did not work on dialysis days, resulting in a substantial portion of this cohort working fewer than 4 days per week. Similar findings have been reported in previous studies, where patients on dialysis, compared to those in the early stages of CKD or kidney transplant recipients, were more often partially work-disabled and on sick leave, particularly on dialysis days [8, 20]. While dialysis therapy can help manage symptoms in advanced CKD and enable patients to remain productive, challenges such as postdialysis fatigue, side effects, and scheduling conflicts often reduce work capacity [7]. These issues are not always adequately addressed, leading to considerable disruptions in employment patterns. This underscores the need for workplace protection for individuals with CKD, including flexible work arrangements, patient-centered dialysis scheduling, and targeted support to help young adults remain employed while undergoing treatment.

Dialysis is an intensive and time-consuming treatment that not only affects the physical health of patients but also significantly disrupts their social and economic lives [21]. In our study, more than half of the unemployed participants had resigned from work due to CKD and dialysis-related challenges. This reflects the broader social impact of hemodialysis, with CKD often described as a “disease of loss” due to its detrimental effects on income, independence, and social identity. For many young adults, leaving employment marks a profound shift in self-worth and increases dependency on family members, further straining household finances. Muehrer et al. reported that poor health insurance coverage, female gender, and age over 49 were associated with a greater likelihood of job loss after initiating dialysis [7]. To address these challenges, a range of interventions, including vocational support, workplace accommodations, supportive healthcare policies, psychosocial care, symptom management, and flexible dialysis modalities, can play a critical role in helping adults on dialysis remain employed and socially engaged [8, 20, 22].

While awareness of kidney transplantation was high among participants, with nearly all acknowledging it as a treatment option, fewer than a third had previously pursued or were actively pursuing transplantation. The most commonly reported obstacles were the absence of a donor and the high cost of the procedure barriers that are particularly burdensome in low-resource settings [12, 23]. This gap highlights the significant challenges young patients face in accessing transplantation, a potentially life-restoring intervention that could enable them to return to productive lives [23]. Without equitable access, many remain dependent on long-term dialysis, which is both physically and emotionally taxing, and financially unsustainable for many [24]. Addressing these barriers through supportive policies, financial assistance, and improved donor programs is essential to improving outcomes and restoring independence for this population.

Comparing unemployment among different forms of kidney replacement therapy (KRT) reveals significant disparities. In Tanzania, unemployment among hemodialysis patients is reported at around 50%, while a study of 68 kidney transplant recipients showed a lower rate of 20%, suggesting better posttransplant work potential [25]. However, access to transplantation is limited, with the program starting in 2017 and performing fewer than 100 procedures to date. In addition, no chronic peritoneal dialysis (PD) program currently exists in the country [5]. As a result, most patients rely on in-center hemodialysis, which continues to interfere with sustained employment [3].

Our study highlights the profound impact of maintenance hemodialysis on the lives of young adults, particularly in relation to employment and socioeconomic stability. Employment among people on dialysis has recently been recognized as a patient-important outcome. Although more than half of the participants remained engaged in work, many experienced significant disruptions, reduced productivity, or were forced to resign due to the physical and logistical demands of dialysis. The burden is further compounded by out-of-pocket treatment costs and limited progress toward kidney transplantation. These findings underscore the urgent need for integrated support systems including flexible work policies, psychosocial support, and improved access to transplantation to help young adults maintain independence, restore productivity, and enhance their overall quality of life.

One key limitation of our study is the absence of a qualitative component, which could have provided deeper insights into the lived experiences of young adults on dialysis. While our quantitative findings highlight the measurable impact on employment and access to transplantation, qualitative data would have enriched our understanding of the personal, emotional, and social dimensions of these challenges. Future studies incorporating in-depth interviews or focus group discussions are essential to capture the nuanced realities behind these numbers and to inform more holistic, patient-centered interventions.

## Figures and Tables

**Table 1:** Demographic and clinical characteristics (*N* = 134).

Variable	*N* (%)
Age	
35 years	65 (48.5%)
35–49 years	69 (51.5%)
Mean age (SD)	35.8 (8.9)
Gender	
Male	75 (55.9)
Female	59 (44.1)
Marital status	
Single	72 (53.7)
Married	59 (44)
Divorced	3 (2.3)
Level of education	
No formal education or primary education	25 (18.6)
Secondary education	49 (36.6)
Tertiary education (college and university)	60 (44.8)
Alcohol use history	
Former alcohol user	23 (17.2)
Never used alcohol	111 (82.8)
Smoking status	
Previous smoker	13 (9.7)
Never smoked	121 (90.3)
Distance to dialysis facility (km)	
0–14.9	73 (54.5)
> 15	61 (45.5)
Median (IQR)	12.5 (8.8–15.0)
Common modality of transport to dialysis	
Public bus	101 (75.4)
Private/rented cars	24 (17.9)
Motor cycles	9 (6.7)
Escort to dialysis	
HD with support	82 (61.2)
HD alone	52 (38.8)
Weekly frequency of hemodialysis	
Once	9 (6.7)
Twice	69 (51.5)
Thrice	56 (41.8)
Duration on hemodialysis therapy (months)	
0–24	75 (56)
> 24	59 (44)
Median (IQR)	19 (9.3–42)
Cause of CKD	
Hypertension	78 (59.1)
Chronic glomerular diseases	24 (18.2)
Unknown	14 (10.6)
Diabetes mellitus	10 (7.6)
Other causes	6 (4.5)
Type of hemodialysis vascular access	
AVF	51 (38.1)
Tunneled catheter	47 (35.1)
Nontunneled catheter	36 (26.9)

**Table 2:** Socioeconomic effects of CKD and dialysis.

Occupational status	
Student	23 (17.2)
Employed	46 (34.4)
Self-employed	32 (23.9)
Unemployed	33 (24.6)
Working on days of dialysis (*N* = 78) (employed or self-employed)	
Yes	43 (55.1)
No	35 (44.9)
Changes in work patterns due to dialysis (*N* = 78) (employed or self-employed)	
Yes	51 (65.4)
No	27 (34.6)
Number of working days per week (*N* = 78) (employed or self-employed)	
≥ 4 days	45 (57.6)
< 4 days	33 (42.4)
Job resignation due to dialysis (*N* = 33) (unemployed)	
Yes	19 (57.6)
No	14 (42.4)
Payment modality for dialysis	
Out of pocket	70 (52.3)
Insurance	64 (47.7)
Awareness of kidney transplant	
Yes	124 (92.5)
No	10 (7.5)
Previously or actively pursuing kidney transplantation	
Yes	37 (27.6)
No	97 (72.4)
Reported obstacle for kidney transplant (*N* = 87)	
Absence of a donor	42 (48.3)
High cost	20 (23)
Both high cost and the absence of a donor	25 (28.7)

## Data Availability

The datasets used and/or analyzed during the current study are available from the corresponding author on reasonable request.

## References

[1] Matsha T. E., Erasmus R. T. (2019). Chronic Kidney Disease in Sub-Saharan Africa. *Lancet Global Health*.

[2] Hariparshad S., Bhimma R., Nandlal L., Jembere E., Naicker S., Assounga A. (2023). The Prevalence of Chronic Kidney Disease in South Africa-Limitations of Studies Comparing Prevalence With Sub-Saharan Africa, Africa, and Globally. *BMC Nephrology*.

[3] Msilanga D. P., Punatar P., Ruggajo P. (2023). Health-Related Quality of Life of Patients Undergoing Haemodialysis Therapy in Dar es Salaam, Tanzania. *African Journal of Nephrology.*.

[4] Mushi L., Krohn M., Flessa S. (2015). Cost of Dialysis in Tanzania: Evidence From the Provider’s Perspective. *Health Economics Review*.

[5] Furia F. F. (2024). Progress in CKD Care and Integration of Adult and Childhood Nephrology Services in Tanzania. *Kidney*.

[6] Silva Junior G. B. (2018). Global Costs Attributed to Chronic Kidney Disease: A Systematic Review. *Revista da Associação Médica Brasileira*.

[7] Muehrer R. J., Schatell D., Witten B., Gangnon R., Becker B. N., Hofmann R. M. (2011). Factors Affecting Employment at Initiation of Dialysis. *Clinical Journal of the American Society of Nephrology*.

[8] Ditschman E. (2021). A Dialysis Patient’s View on Dialysis Employment Loss. *Clinical Journal of the American Society of Nephrology*.

[9] Niang A., Lemrabott A. T. (2020). Global Dialysis Perspective: Senegal. *Kidney*.

[10] Luyckx V. A., Stanifer J. W. (2018). Global Burden of Kidney Disease. *Bulletin of the World Health Organization*.

[11] Furia F. F., Shoo J. G., Ruggajo P. J. (2024). Establishing Kidney Transplantation in a Low-Income Country: A Case in Tanzania. *Replace*.

[12] Hasan R. S. A., Anwar Naqvi S. A., Ahmed E. (2005). Renal Transplantation in Developing Countries. *Kidney Diseases in the Developing World and Ethnic Minorities*.

[13] Karimi M., Brazier J. (2016). Health, Health-Related Quality of Life, and Quality of Life: What is the Difference?. *PharmacoEconomics*.

[14] Msilanga D., Muiru A., Balandya E., Liu K. (2024). Point of Care Creatinine Testing for Early Detection of Renal Dysfunction in Tanzanian HIV Patients: A Study Protocol. *BMC Nephrology*.

[15] Findings K. (2022). The United Republic of Tanzania the Republic of Korea People’s Republic of China.

[16] Haji M. Youth Employment in Tanzania Taking Stock of the Evidence and Knowledge Gaps About the International Development Research Centre.

[17] Msilanga D., Shoo J., Mngumi J. (2024). Patterns of Vascular Access Among Chronic Kidney Disease Patients on Maintenance Hemodialysis at Muhimbili National Hospital. A Single Centre Cross-Sectional Study. *PLOS Global Public Health*.

[18] J Meremo A., B Masalu M., Sabi I. (2018). Prevalence and Risk Factors Associated With Chronic Kidney Disease Among Patients Presenting at a Haemodialysis Unit in Dodoma, Tanzania. *East African Health Research Journal*.

[19] Ghazi H. F. Sample Size Made Easy.

[20] Alma M. A., van der Mei S. F., Brouwer S. (2022). Sustained Employment, Work Disability and Work Functioning in CKD Patients: A Cross-Sectional Survey Study. *Journal of Nephrology*.

[21] Mousa I., Ataba R., Al-ali K., Alkaiyat A., Zyoud S. H. (2018). Dialysis-Related Factors Affecting Self-Efficacy and Quality of Life in Patients on Haemodialysis: A Cross-Sectional Study From Palestine. *Replace*.

[22] Essue B. M., Wong G., Chapman J., Li Q., Jan S. (2013). How are Patients Managing With the Costs of Care for Chronic Kidney Disease in Australia? a Cross-Sectional Study. *BMC Nephrology*.

[23] Awuah W. A., Ng J. C., Bulut H. I. (2023). The Unmet Need of Organ Transplantation in Africa. *International Journal of Surgery*.

[24] Akoh J. A. (2011). *Renal Transplantation in Developing Countries*.

[25] Shoo J., Msilanga D., Mngumi J. (2024). Clinical Profile and Outcome of Kidney Transplantation at Muhimbili National Hospital, Tanzania. *BMC Nephrology*.

